# Physically-Based Reduced Order Modelling of a Uni-Axial Polysilicon MEMS Accelerometer

**DOI:** 10.3390/s121013985

**Published:** 2012-10-17

**Authors:** Aldo Ghisi, Stefano Mariani, Alberto Corigliano, Sarah Zerbini

**Affiliations:** 1 Dipartimento di Ingegneria Strutturale, Politecnico di Milano, Piazza Leonardo da Vinci 32, 20133 Milano, Italy; E-Mails: stefano.mariani@polimi.it (S.M.); alberto.corigliano@polimi.it (A.C.); 2 MSH Division, STMicroelectronics, Via Tolomeo 1, 20010 Cornaredo, Italy; E-Mail: sarah.zerbini@st.com

**Keywords:** MEMS inertial sensors, reduced order modelling, shocks and drops, polysilicon

## Abstract

In this paper, the mechanical response of a commercial off-the-shelf, uni-axial polysilicon MEMS accelerometer subject to drops is numerically investigated. To speed up the calculations, a simplified physically-based (beams and plate), two degrees of freedom model of the movable parts of the sensor is adopted. The capability and the accuracy of the model are assessed against three-dimensional finite element simulations, and against outcomes of experiments on instrumented samples. It is shown that the reduced order model provides accurate outcomes as for the system dynamics. To also get rather accurate results in terms of stress fields within regions that are prone to fail upon high-*g* shocks, a correction factor is proposed by accounting for the local stress amplification induced by re-entrant corners.

## 1. Introduction

Several attempts to provide efficient, robust and accurate (or, at least, informative) reduced order models (ROMs) for nonlinear systems, with a specific focus on MEMS, have been recently published. Accounting for the nonlinearities arising from the coupled electro-mechanical, or even electro-thermo-mechanical physics governing the system behaviour, methodologies to define Krylov subspaces and therefore reduce the computational costs of the analyses were proposed in [1]. Typically, such procedures were centred around Lanczos or Arnoldi's methodologies, see e.g., [2–6]. When nonlinearities of the model are weak, adaptive procedures for the projection of the governing equations onto the reduced order space, within which the system is mathematically assumed to evolve, may prove sufficient [7]. Instead, when nonlinearities are strong, Taylor series expansions or piecewise-linearisations were shown to be necessary in [2,8] to attain accuracy. Reviews of the current state of the art can be found, e.g., in [9,10]. Moreover, an interesting classification of the methodologies and an assessment of their performances in the presence of squeeze-film fluid and thermo-elastic dampings were reported in [11,12].

Proper orthogonal decomposition (POD) methodologies were also adopted to reduce the order of MEMS modelling [13–15]. Like the previously mentioned approaches, POD was developed for model order reduction of linearly evolving time-invariant systems, see e.g., [16]. Since this methodology consists in capturing snapshots of the system response to the external actions during an initial training stage of the analysis, such stage has to provide enough information concerning the nonlinearities affecting the sensor behaviour [17]. The training stage also has to provide information concerning a possible time-varying physics of the problem. This turns out to be a very challenging task, since loading conditions can sudden change and a forecast of the effects of this change on the system condition proves difficult. One of the methodologies typically adopted in such cases is based on a collection of new snapshots once the accuracy of the ROM gets degraded. Obviously, this additional stage reduces the computational gain and *ad-hoc* techniques are required to balance speed-up and accuracy.

Physically based ROMs of the mechanical behaviour of microsystems were instead presented in [18,19]. In these papers, instead of considering the problem in an abstract way and then operating through mathematical tools to project the system dynamics onto subspaces of the actual evolution one, the mechanics of the movable parts of the sensors as well as the effect of external actions on them were taken account of. While this approach can prove very effective, it cannot be applied generally since it must be finely tuned or adapted to every specific geometry or layout.

Focusing on the physical effects of shocks and drops on micro-inertial sensors, it can be shown that the mechanical side of the problem is by far the most prominent one; the electrical side can be instead disregarded. This is basically linked to the high levels of acceleration induced by the shock loading: in [20] these levels were shown to exceed 10^5^ *g*, *g* being the gravity acceleration. Because of the constrained motion of the movable parts of the MEMS inside the cavity between die and cap, inertial and possible contact forces turn out to exceed by orders of magnitude the electrical ones. Hence, in the aforementioned situations only the structural dynamics of movable parts need to be reduced in order.

Because of the large diffusion of MEMS devices in commercial applications for consumer electronics, the post-impact response of polysilicon micro-accelerometers has received attention in the recent years [20,21]. In fact, shocks still represent an important issue as for the reliability of inertial micro-devices; failure linked to micro-crack spreading in high stressed regions of the MEMS can suddenly occur [22]. Because of the complexity of the problem for packaged devices, involving phenomena that take place over various length-scales (ranging from the order of mm, down to the order of nm), the actual mechanical behaviour of the MEMS affected by these exceptional events is yet to be fully understood.

In a series of recent papers [23–26], we accounted for the whole physics of possible failure processes of the polysilicon film that constitutes the movable parts of MEMS accelerometers, through a one-way (or uncoupled [27]) top-down approach. We developed separate models at the macro-scale (or package level), at the meso-scale (or sensor level), and at the micro-scale (or polysilicon level). Readers can find a thorough discussion on the offered methodology in [28]; here, it suffices to mention that, according to the distinction among the three length-scales, a different major phenomenon is simulated at each level. At the macro-scale the focus is on the propagation of stress waves inside the package; at the meso-scale the focus is instead on the dynamics of the movable parts of the whole MEMS; eventually, at the micro-scale the degradation of the mechanical properties of the polysilicon film, and the resulting failure mechanism are simulated. While this three-scale approach proved accurate and allowed us to match also unexpected effects linked to the package in case of drops [29], it turned out to be time-consuming. We therefore investigated procedures to reduce the computational costs. First, in [30,31] we developed micromechanically-informed constitutive models for the polysilicon film to be adopted at the meso-scale, so as to avoid the micro-scale analyses resting on a Monte Carlo procedure to account for the statistical fluctuations (in space and from sensor to sensor) of crystal morphology. Second, in [32,33] we started assessing ROMs for the whole sensors, so as to speed up also the meso-scale analyses. Accounting for the fact that shocks induce wild oscillations of the movable parts of inertial sensors, which need nonlinear dynamic analysis because of contact with die and cap surfaces, and observing that the vibrations of the seismic plate can be disregarded when compared to the vibrations of the suspension springs, a physically- or mechanically-based ROM was developed.

In [32] we compared the outcomes of a ROM, obtained through the aforementioned approach, with experimental data collected during laboratory drop tests on packaged uni-axial MEMS accelerometers. We showed that the model is capable of matching the actual MEMS dynamics, but we did not discuss its accuracy at varying maximum acceleration levels under guided (namely, smooth) or free falls and the computational gain with respect to finite element (FE) simulations. In this paper, we therefore focus on these two issues to validate the methodology and the ROM itself. The remainder of the paper is hence organised as follows. Section 2 provides a description of the geometry and expected working conditions of the investigated commercial device, along with the assumptions and related equations governing the proposed ROM. Section 3 collects the results linked to two different test conditions, respectively termed low-*g* and high-*g* on the basis of the attained maximum level of acceleration felt by the sensor. Finally, conclusions and possible future model enhancements are discussed in Section 4.

## 2. Reduced Order Modelling of a Uni-Axial MEMS Accelerometer

**Figure 1.**
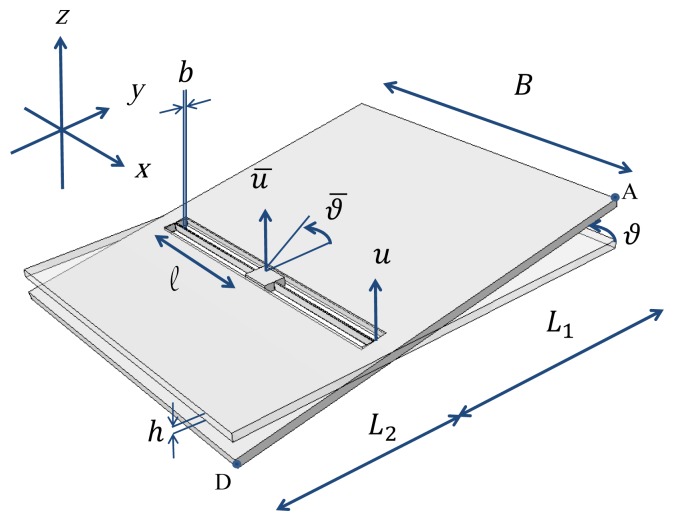
Geometry of the uni-axial MEMS accelerometer, and notation.

Let us consider the commercial off-the-shelf, uni-axial MEMS accelerometer depicted in Figure 1. This accelerometer is part of a three-axis one (full details are provide in [34]) constituted also by a bi-axial one, measuring in-plane accelerations (along the *x* and *y* axes); the considered uni-axial one measures instead the out-of-plane acceleration (along the z axis). We focus here on the uni-axial mechanical device only, since it is more prone to failure than the bi-axial sensor due to its layout. Thanks to the very small cross-talk of this family of MEMS accelerometers, see [35], accelerations along the *z* axis do not induce reliability issues on the other moving and/or sensing parts.

The uni-axial accelerometer is designed to sense accelerations normal to the substrate with a target sensitivity of about 0.65 V/*g* and deviation from linearity of 0.6% of the full scale, up to a maximum value of ±2 *g*. The rather standard layout of the movable parts is constituted by a massive seismic plate, whose side lengths are *B* = 660 *μ*m and *L* = *L*_1_ + *L*_2_ = 760 *μ*m, connected to the central support through two slender beams of length *ℓ* = 259 *μ*m and width *b* = 2.6 *μ*m. The common thickness of plate and beams is *h* = 15 *μ*m. To allow the release of movable parts from the substrate during the etching phase (see [36]), the plate features a regular pattern of etch access holes which reduce its mass proportionally. When the seismic plate is exposed to out-of-plane accelerations, the supporting beams are deformed torsionally; tilting of the plate then induces a variation of the capacitance, proportional to the external action within the working regime. Due to the spring slenderness, beams might buckle in case of in-plane accelerations; this is prevented through stoppers (not shown in the picture) that constrain the lateral motion of the plate.

**Figure 2.**
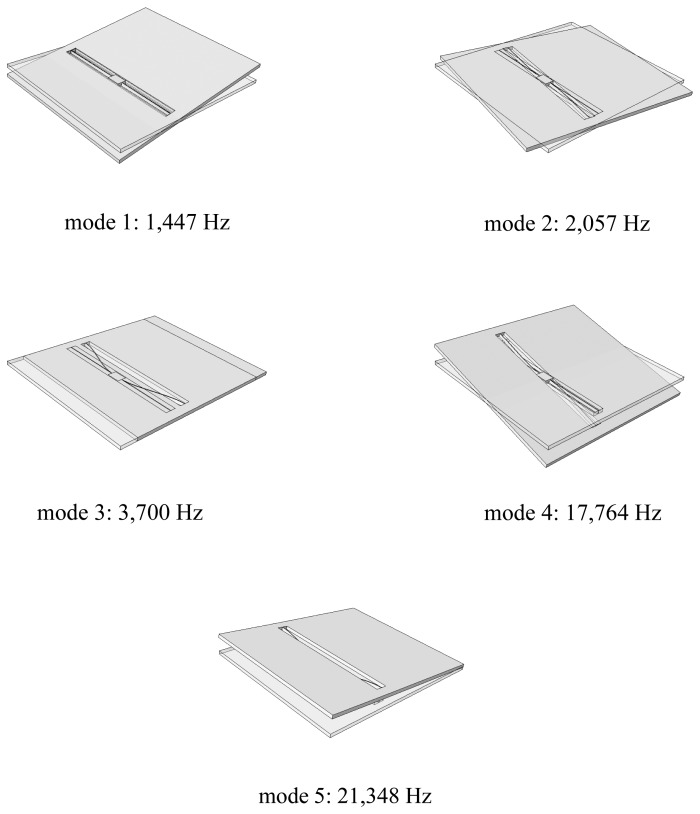
First five vibration modes of the uni-axial accelerometer, and relevant resonance frequencies.

Comparison FE results have been obtained with the commercial Abaqus code (Simulia) [37]. By means of a three-dimensional model of the device, featuring 35,682 nodes, 28,768 elements and more than 100,000 degrees of freedom (DOFs), the vibration modes of the sensor have been identified, along with the relevant frequencies. The first five modes, featuring lower frequencies, are depicted in Figure 2; here, for clarity of presentation, etch holes in the plate have been removed from the plots. As stated in [34], the resonance frequency of the fundamental, working mode is around 1.5 kHz; as mentioned before, such mode consists in a torsional deformation of the springs, while the plate behaves as a rigid body. The subsequent modes #2 and #3 are not relevant in this investigation, since they would involve the aforementioned constrained, in-plane motion of the plate. Moreover, they are not excited by the considered external actions since the device is sensitive to out-of-plane accelerations only. Mode #4 is instead anti-symmetric about the central anchor point: with a focus on the vertical acceleration, assumed to be constant in space because of the small size of the plate, this mode is not excited as well. Mode #5, whose frequency is higher than 21 kHz (to be compared to 1.5 kHz of the working mode), features a coupled torsional and out-of-plane bending deformation mechanism of the two springs, whereas the plate still gets displaced like a rigid body. Higher-frequency modes, not reported here for brevity, share some characteristics with modes #2, #3 and #4, and are therefore not excited by out-of-plane accelerations. Moreover, vibrations of the seismic plate are not displayed by the sensor until frequencies of about 200 kHz. All the above discussed mode shapes and vibration frequencies have been obtained by considering the following elastic properties of the polysilicon film [38,39]: *E_x_* = *E_y_* = 152.9 GPa, *E_z_* = 130.1 GPa, *ν_xy_* = 0.2, *ν_xz_* = *ν_yz_* = 0.28, *G_xz_* = *G_yz_* = 79.6 GPa. Such transversely isotropic moduli were obtained in [23], accounting for the polycrystalline morphology of the film and for the FCC crystal lattice of each silicon grain, and then compared to analytical bounds in [40] to ascertain their accuracy, see also [41].

By assuming the external acceleration field to be upper bounded by 200 kHz, see also [32], we can start building the ROM for the whole sensor by neglecting plate deformations. According to what is depicted in Figure 2, we therefore have to consider only torsional and out-of-plane bending deformations of the two suspension springs; the relevant two DOFs of the ROM are then the (absolute) plate rotation *ϑ* and displacement *u*, see Figure 1. In a relative frame moving with the anchor, the actual deformation of the springs is captured by the rotation and displacement variations, respectively denoted as Δ*ϑ* = *ϑ* − *ϑ̄* and Δ*u* = *u* − *ū*, where the overbar denotes the assigned, time-varying values at anchor. The restoring (elastic) torque and out-of-plane force in the beams respectively read:

(1) $$\begin{array}{cc} M_{e} & =2k_{t}\Delta \vartheta \\ F_{e} & =2k_{b}\Delta u \end{array}$$

where the coefficient 2 accounts for the two suspension springs working in parallel. In Equation (1), $k_{t}=\frac{G_{xz}I_{t}}{\ell }$ is the torsional stiffness and $k_{b}=\frac{12E_{x}I_{b}}{\ell^{3}}$ is the bending stiffness of a single beam of length *ℓ* and cross-section dimensions *b* and *h*; hence, the relevant moments of inertia are, respectively, *I_t_* = *κhb*^3^ (where *κ* = 1/3.43) and $I_{b}=\frac{bh^{3}}{12}$, see [42]. In these formulae, the values of the elastic moduli *G_xz_* and *E_x_* are those introduced above.

Inertial terms arising from the assumed kinematics are:

(2) $$\begin{array}{c} M_{i}=\int_{\Omega }\rho y\left[\ddot{\bar{u}}+\Delta \ddot{u}+y\left(\ddot{\vartheta }+\Delta \ddot{\vartheta }\right)\right]d\Omega =M_{u\vartheta }(\ddot{\bar{u}}+\Delta \ddot{u})+M_{\vartheta \vartheta }\left(\ddot{\bar{\vartheta }}+\Delta \ddot{\vartheta }\right)\\ F_{i}=\int_{\Omega }\rho \left[\ddot{\bar{u}}+\Delta \ddot{u}+y\left(\ddot{\vartheta }+\Delta \ddot{\vartheta }\right)\right]d\Omega =M_{uu}(\ddot{\bar{u}}+\Delta \ddot{u})+M_{u\vartheta }\left(\ddot{\bar{\vartheta }}+\Delta \ddot{\vartheta }\right) \end{array}$$

being:

(3) $$\begin{array}{ccc} M_{uu}=\int_{\Omega }\rho d\Omega & M_{u\vartheta }=\int_{\Omega }\rho yd\Omega & M_{\vartheta \vartheta }=\int_{\Omega }\rho y^{2}d\Omega \end{array}$$

In Equation (2), the superimposed dots stand for time derivative, and integration is performed over the plate volume Ω disregarding the small contributions provided by the springs. Moreover, *ϑ̄̈* and *ṻ* respectively represent the rotational and translational accelerations of the anchor, and *ρ* is the scaled (because of the holed geometry) mass density of the polysilicon plate.

The system dynamics, accounting also for proportional damping, is therefore governed in the ROM by the following two coupled equations of motion:

(4) $$\left[\begin{array}{cc} M_{uu} & M_{u\vartheta }\\ M_{u\vartheta } & M_{\vartheta \vartheta } \end{array}\right]\;\left[\begin{array}{c} \Delta \ddot{u}\\ \Delta \ddot{\vartheta } \end{array}\right]+\left[\begin{array}{cc} D_{uu} & D_{u\vartheta }\\ D_{u\vartheta } & D_{\vartheta \vartheta } \end{array}\right]\;\left[\begin{array}{c} \Delta \dot{u}\\ \Delta \dot{\vartheta } \end{array}\right]+\left[\begin{array}{cc} 2k_{b} & 0\\ 0 & 2k_{t} \end{array}\right]\;\left[\begin{array}{c} \Delta u\\ \Delta \vartheta \end{array}\right]=-\left[\begin{array}{cc} M_{uu} & M_{u\vartheta }\\ M_{u\vartheta } & M_{\vartheta \vartheta } \end{array}\right]\;\left[\begin{array}{c} \ddot{u}\\ \ddot{\vartheta } \end{array}\right]$$

or briefly:

(5) $$\text{M}\Delta \ddot{\chi }+\text{D}\Delta \dot{\chi }+\text{K}\Delta \chi =-\text{M}\ddot{\bar{\chi }}$$

where Δ***χ*** = {Δ*u* Δ*ϑ*}*^T^* and ***χ̄̈*** = {*ṻ ϑ̄̈*}*^T^*. Since entries of mass and damping matrices can be different by orders of magnitude, Equation (5) is numerically handled as:

(6) $$\Delta \ddot{\chi }+M^{-1}D\Delta \dot{\chi }+M^{-1}K\Delta \chi =-\ddot{\bar{\chi }}$$

through pre-multiplication by ***M***^−l^. Accounting for proportional damping, we set ***D*** = *μ****M*** (hence ***M***^−1^ ***D*** = *μ****I***) where *μ* = *w*/*Q*, *Q* is the expected quality factor under such loading conditions and *w* = 9.091 kHz is the circular frequency of the fundamental mode. As already remarked in [32], since Δ*u* and Δ*ϑ* are dimensionally inhomogeneous and take numerical values in very different ranges, a re-normalisation (or re-scaling) of them can help in assuring stability of the solution.

During oscillations, plate corners may get into contact with the die and cap surfaces. Algorithmically, variables Δ*u* and Δ*ϑ* are constrained to take values in the following ranges:

(7) $$\begin{array}{c} -gd+\frac{h}{2}\leq \Delta u+L_{1}\tan(\Delta \vartheta )\leq g_{c}-\frac{h}{2}\\ -gd+\frac{h}{2}\leq \Delta u-L_{2}\tan(\Delta \vartheta )\leq g_{c}-\frac{h}{2} \end{array}$$

where (see also Figure 1): $\frac{h}{2}$ is included to account for the thickness of the moving plate (it represents the distance of the top and bottom plate corners from its mid-plane); *g_d_* = 1.8 *μ*m and *g_c_* = 15.8 *μ*m are, respectively, the gaps between plate corners and die and cap surfaces. During the analyses, contact is accounted for through a so-called penalty formulation: if relations (7) are all satisfied as inequalities, the stiffness matrix ***K*** previously described is adopted to advance the solution in time; if instead at least one of relations (7) is satisfied as an equality, the entries of the stiffness matrix are proportionally amplified as ***K̃*** = *ψ****K*** to prevent (or, at least, to keep small) the penetration between plate and die, or plate and cap. The penalty coefficient *ψ* is usually strongly dependent on the time discretization, the kind of input loading and the geometry of the vibrating structure. While in principle energy balance considerations allow to estimate *ψ*, in practice it is set empirically so as to efficiently and robustly carry out the calculations; in this work, a value *ψ* = 750 has been used in all the simulations.

As far as the solution of governing Equation (6) is concerned, a direct time integration scheme has been adopted. To damp possible spurious high frequency oscillations not arising from the real physics of the problem, the *α*-method has been implemented, see [43]. To ensure unconditional stability and second-order accuracy while contact conditions (7) do not play a role, algorithmic parameters were set as follows (see also [44]): *α* = −0.05, *β* = 0.275625, *γ* = 0.55.

## 3. Results

**Figure 3.**
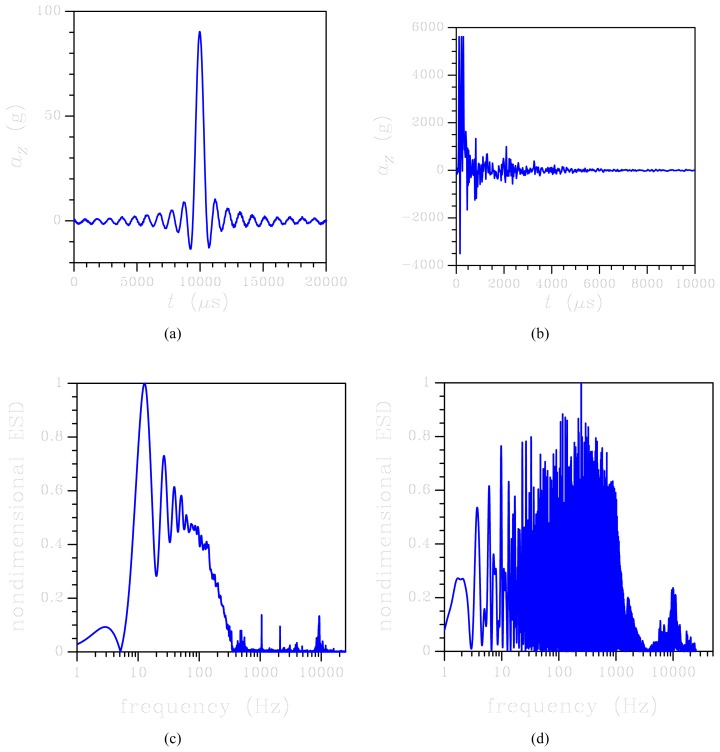
Shock tests. Top row: acceleration histories felt by the sensor during the (**a**) low-*g* and (**b**) high-*g* experiments. Bottom row (**c**,**d**): energy spectral density (ESD) obtained through a Fourier transform of the relevant input accelerations.

In this Section, the ROM accuracy and performance (in terms of reduction of the computational costs) are assessed against FE simulations and available experimental data. Both the ROM and the FE model are fed by the input acceleration loadings depicted in Figure 3, already considered in [32]. The two conditions differ in terms of maximum acceleration peaks; accordingly, we refer to loading in Figure 3(a) as the low-*g* one (even though the peak of around 90 *g* by far exceeds the working conditions); we instead refer to loading in Figure 3(b) as the high-*g* one (with a peak value of about 5,500 *g*). Besides peak value, the two histories also differ as for the duration of the acceleration pulse and, on the top of all, as for the kind of time evolution: in the low-*g* case, a sinusoidal-like smooth variation was induced by an electrodynamic shaker; in the high-*g* case, a wild non-smooth variation was experimentally induced by a free-fall in an impact tester. This explains the two very different contents of excited frequencies, as shown in Figure 3(c,d). In the two testing setups, the MEMS was mounted on a support board to drive the output to a data acquisition card, so as to measure the output voltage during the test. The board was rigidly connected to a massive brass plate, featuring a reference quartz accelerometer to provide the input acceleration history.

Both the loading conditions depicted in Figure 3 feature null rotational accelerations *ϑ̄̈* of the testing device and, therefore, of the MEMS die if deformations of the support board are disregarded.

In the comparison FE analyses, according to the ROM, a proportional damping has been considered, with the same quality factor ***Q*** and circular frequency *w* of the fundamental (tilting) vibration mode. Since vibrations of the movable parts can be experimentally monitored only through the time evolution of the sensor output voltage, and since saturation in the output signal occurs because of the plate contact with die and cap out of the sensor working range, the FE simulations help to assess the accuracy of the ROM results through: impact-induced motion of the plate; stress state in the supporting springs, possibly prone to fail in case of high-*g* shocks. Moreover, nonlinearities in the system response, obviously disregarded under working conditions, can be assessed through comparison with the experimentally acquired signals. In doing this, the variation of the output voltage is numerically computed as:

(8) $$\Delta V(t)=\bar{V}+\zeta \Delta a(t)$$

where *V̄* is the supply voltage, *ζ* is the device sensitivity, and Δ*a* is the variation of the out-of-plane acceleration with respect to the gravity one.

**Figure 4.**
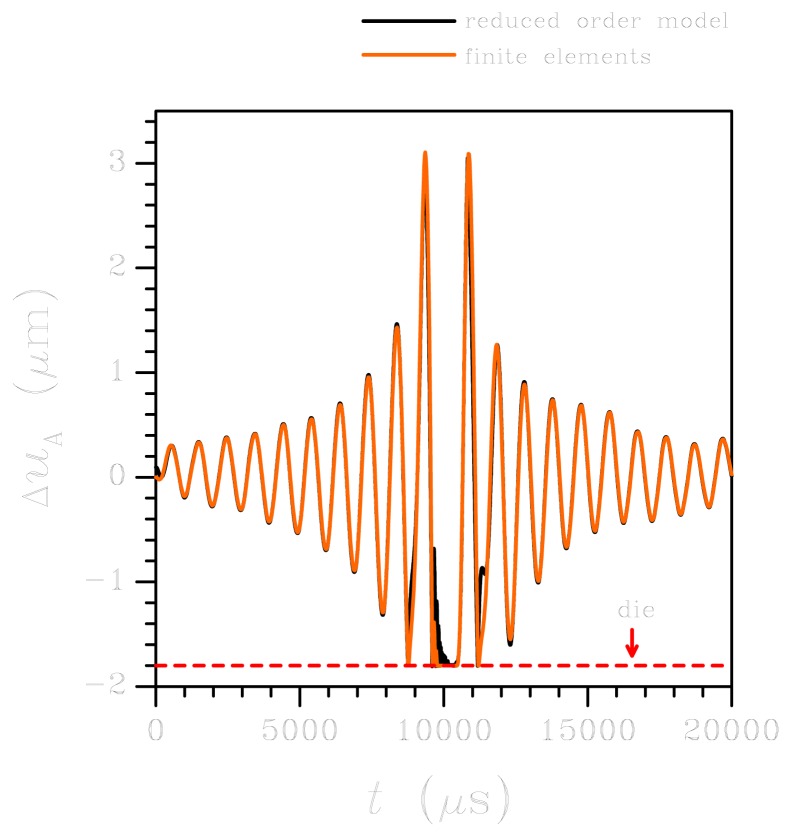
Low-*g* test: time evolution of the relative displacement Δ*u_A_* at the plate corner A (see Figure 1). Comparison between FE and ROM results.

Let's start by considering the low-*g* test. Figure 4 shows the time history of the relative (between plate and die) out-of-plane displacement at corner A, as obtained via ROM and FE analyses; similar results are obtained as for the displacement at corner D. Besides the noteworthy agreement between the two models, which basically validates the hypothesis of rigid plate behaviour, it is interesting to focus on the solution around *t* = 10,000 *μ*s when the plate strikes the die surface more than once (represented by the dashed horizontal line in the graph).

**Figure 5.**
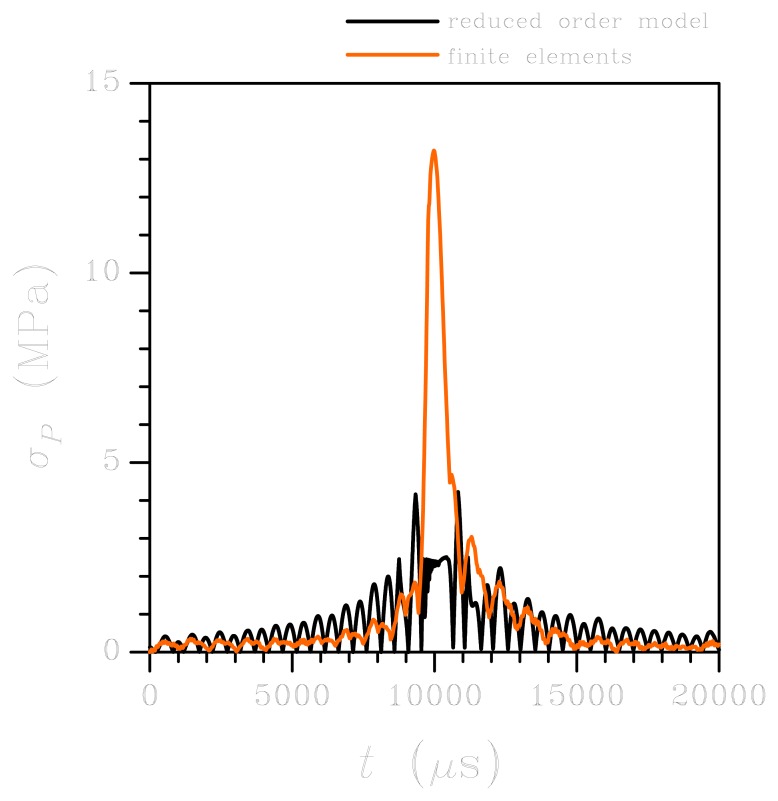
Low-*g* test: time evolution of the maximum principal stress in the plate-spring connection region. Comparison between FE and ROM results.

Because of the spectral density of the input in this low-*g* case, upper bounded by a frequency of about 10 kHz (see Figure 3(c)), only torsional vibrations of the springs are excited: this is clearly evidenced by the vibrations in Figure 4 with a period of about 900 *μ*s, corresponding to this type of deformation (mode #1 in Figure 2). As far as the reliability of the sensor subject to shock-like loadings is concerned, Figure 5 reports the evolution of the maximum principal stress *σ_P_* in the springs. Here *σ_P_* is computed as the envelope of the principal stresses in the whole spring; because of the re-entrant corners at the connection with the anchor and the seismic plate, this maximum is actually located in critical regions very close to the end cross-sections of the supporting beams, see [28]. The value of stress *σ_P_* is considered the triggering one for possible failure mechanisms, because of the overall brittle properties of polysilicon; according to a Rankine-like description of dissipative mechanisms (like cracking formation) in brittle materials, the maximum principal stress has to be monitored (see [31]). The reported values for the ROM represent the current envelope, as induced by torsion and bending (even if the second contribution may be negligible), computed according to the theory of anisotropic, slender beams [42]. Such theory obviously misses contributions linked to the re-entrant corners at both ends of each spring; this explains the difference, in terms of peak values around *t* = 10,000 *μ*s, between the ROM results and the FE solution (which instead accounts for that). The time evolution of *σ_P_* is differently predicted by the two models; this outcome is due to the assumed kinematics of the ROM, which does not appropriately describe what happens in those regions when plate corners hit the die surface and lead to the generation of stress waves that eventually impinge upon the re-entrant corners and cause local interaction effects that change the stress field.

**Figure 6.**
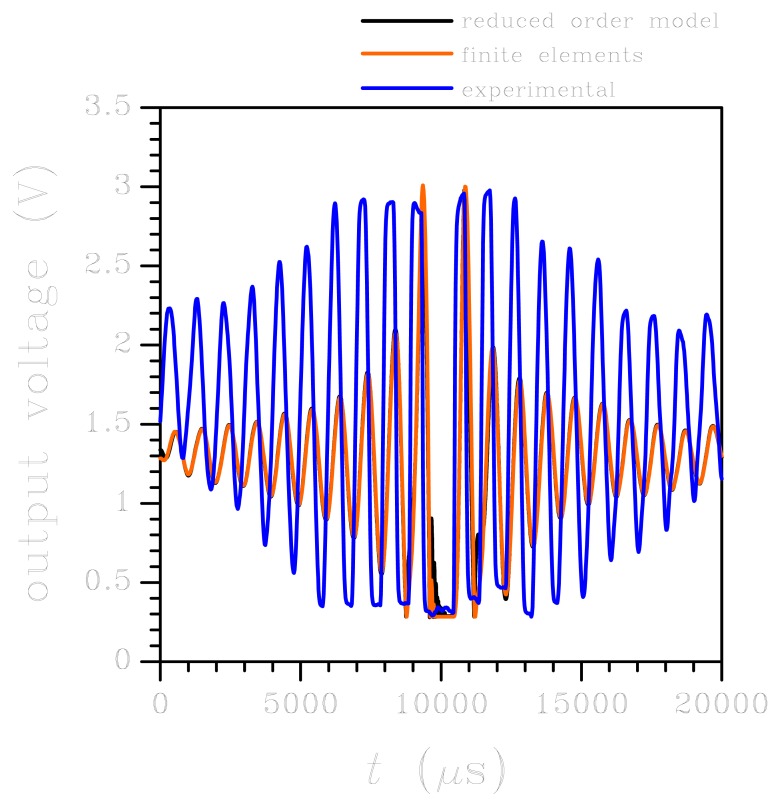
Low-*g* test: time evolution of MEMS output. Comparison between experimental data and numerical results.

When the output voltage evolutions obtained with the two numerical approaches are compared with the experimental data, see Figure 6, it appears that the period of oscillations is well matched, with a small drift linked to the disregarded vibrations of the board. While the electro-mechanical coupling can be considered not to affect the system dynamics around *t* = 10,000 *μ*s, it may play a role well before and after the central portion of the reported output voltage, when the external acceleration level does not exceed 5*g* (see Figure 3) and is therefore comparable to the full scale of the sensor; this can further explain the shift in time somehow visible in Figure 6. However, the main difference between numerics and experimental is reported in terms of amplitude of the oscillations. This discrepancy is linked to two causes, basically (1) we have disregarded board deformations, which actually enhance sensor dynamics by adding an additional compliance to the testing apparatus, and (2) we have assumed the output voltage to be linearly linked to the motion of the seismic plate and, therefore, to the input acceleration according to Equation (8), where the coefficient *ζ* is the one holding within the working regime. Out of the working regime, the linearisation of the sensor input-output relation is not accurate anymore, and nonlinearities affecting the *V* = *V*(*a*) should be appropriately defined through testing, if possible.

**Figure 7.**
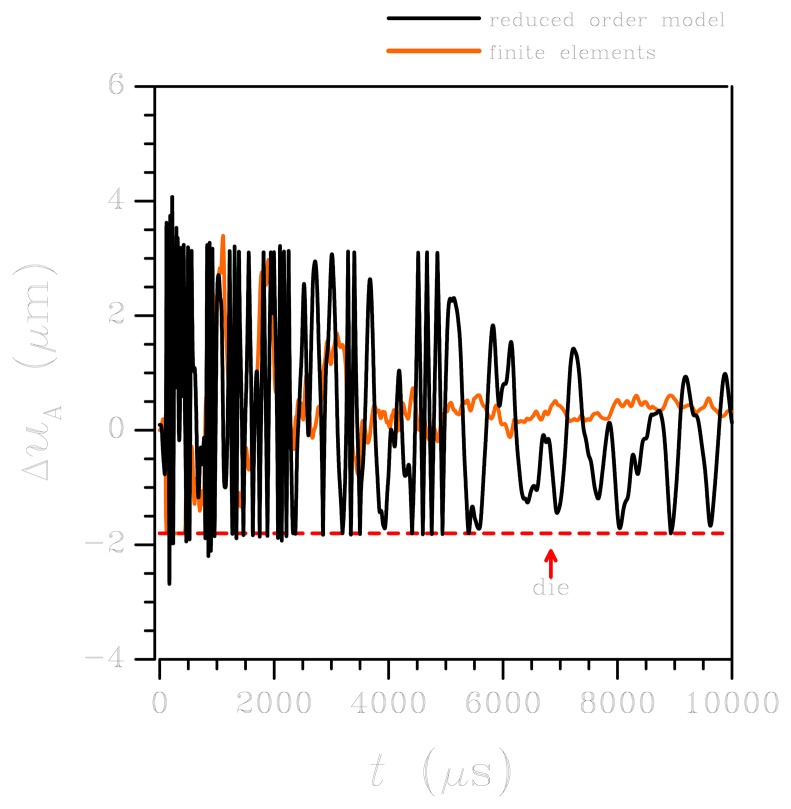
High-*g* test: time evolution of the relative displacement Δ*u_A_* at the plate corner A (see Figure 1). Comparison between FE and ROM results.

**Figure 8.**
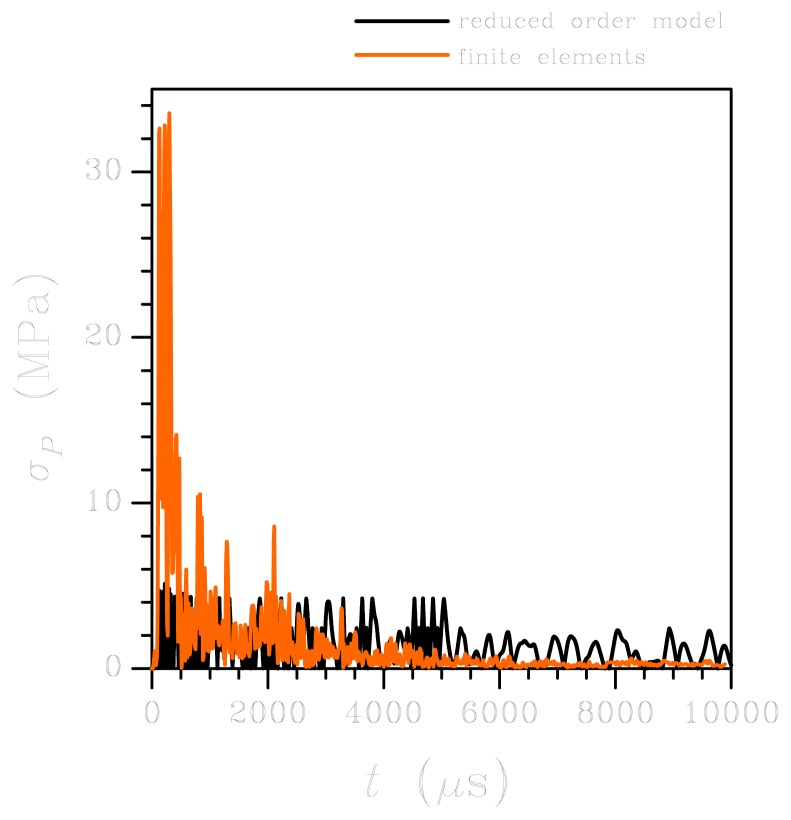
High-*g* test: time evolution of the maximum principal stress in the plate-spring connection region. Comparison between FE and ROM results.

Let's move now to the high-*g* case. The sensor response in terms of relative out-of-plane displacement at corner A is depicted in Figure 7: the ROM and FE results are reported to be in fairly good agreement up to 2,000 *μ*s; then the FE solution seems to overestimate the effect of damping, as the oscillations get reduced much in amplitude. This effect can be partially explained by considering the spectral density of the high-*g* input, see Figure 3(d): at variance with the low-*g* case, higher-order vibration modes are excited since the spectrum is not upper bounded by a frequency of 10 kHz. Accordingly, input energy can be driven to excite vibrations of the plates, thereby reducing the amount of energy available to dynamically deform the suspension springs. The energy transfer also affects the damping of the system, which cannot be described appropriately by considering the circular frequency *w* of the fundamental deformation mode. The same kind of discrepancy between ROM and FE results is also reported in Figure 8, in terms of maximum principal stress *σ_P_* in the critical regions introduced above for the low-*g* case: beyond *t* = 2,000 *μ*s, the over-damped FE solution lead to an almost vanishing stress field. Before that threshold, the ROM underestimates *σ_P_*, in accordance with what is reported for the low-*g* case; once again, disregarding the local effects of re-entrant corners in the ROM solution does not allow to match the FE outcomes.

**Figure 9.**
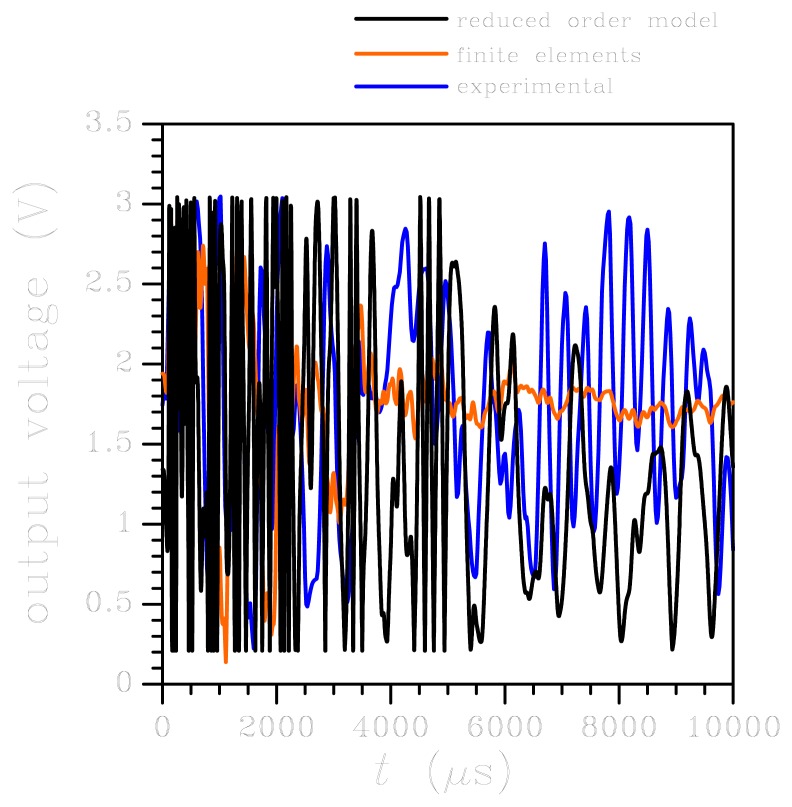
High-*g* test: time evolution of MEMS output. Comparison between experimental data and numerical results.

Because of the wild oscillations of springs and plate induced by the high-*g* test, leading to multiple impacts against the die surface during the whole analysis (see Figure 7), a major frequency or period of oscillations can not be recognised in the solution. Anyhow, possible shifts in the response caused by disregarding the electro-mechanical coupling are here expected not to play a role because of the very high acceleration levels reported in Figure 3(b). This is shown in Figure 9, where the ROM output appears in good agreement with the available experimental time evolution of voltage. Once more, the FE analysis is reported to provide too much damping that causes a sudden reduction of the voltage beyond *t* = 2,000 *μ*s. In a much more evident fashion than the low-*g* case, signal saturation prevents any detailed experimental-numerical comparison. At this point it is worth mentioning that the goal of the original experimental testing campaign, reported in [32], was not to provide a validation of the ROM; contrarily, the ROM was built in order to understand sensor dynamics under shock loading conditions so as to establish critical thresholds for MEMS reliability. What turned out from those experiments is that standard sensors, like the one here considered, can sustain high-*g* acceleration levels without malfunctioning, since the stress field in critical regions results to be much below the characteristic tensile strength of polysilicon (typically higher than 1 GPa).

There are now three basic issues to discuss. First, we have to show the computational gain obtained by running the ROM instead of the FE analyses, keeping the accuracy aside (in this regard, see the discussions above and to follow). The speedups relevant to the two test cases are reported in Table 1, to show that the low-*g* case can be fast simulated through the ROM; here speedups are computed as the ratio between the CPU time required by the FE analysis and the CPU time required by ROM analysis, on a Intel(R) I7 2.7 GHz, 8 GB RAM personal computer. The lower speedup related to the high-*g* case can be explained in this way: when contact conditions (7) hold, the time step size adopted to advance in time the solution of the ROM equations of motion is reduced to $\frac{1}{10000}$ of the initial value (in the case here reported moving from 0.1 *μ*s to 10^−5^ *μ*s) to guarantee the stability of the solution.

**Figure 10.**
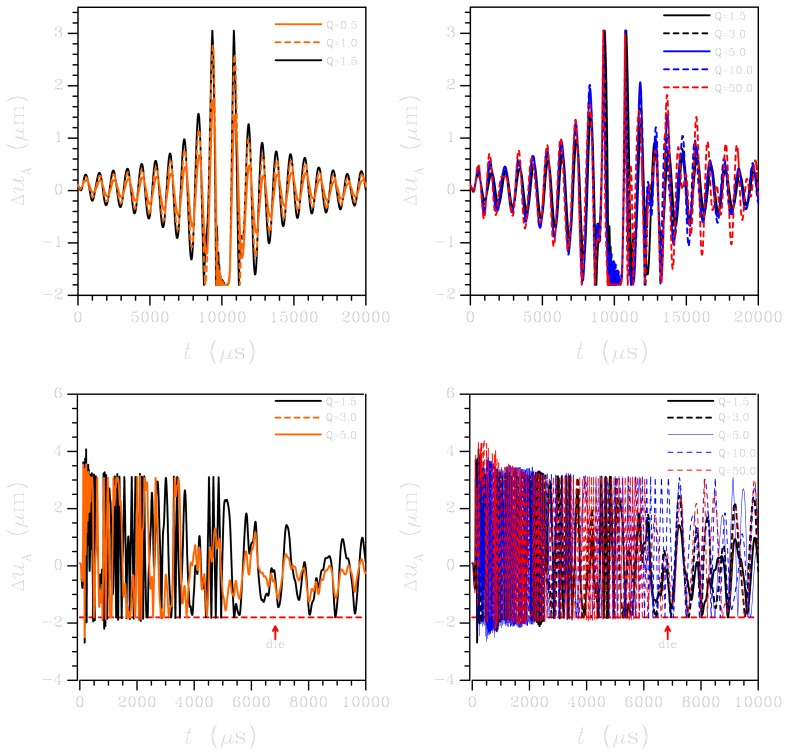
Reduced order modelling: effect of damping on the (top) low-*g* and (bottom) high-*g* MEMS responses, in terms of relative displacement Δ*u_A_*.

Second, we check how the quality factor *Q* in Equation (6) is affecting the solution of the ROM. Thanks to the speedup guaranteed by the ROM over the FE simulations, a parametric analysis to investigate how the output voltage is changed by *Q* turns out to be affordable, in terms of computational costs. Figure 10 shows the results of such parametric analysis, concerning both the low-*g* test (top row) and the high-*g* test (bottom row). In the graphs, *Q* is varied in the range 0.5–50. In the low-*g* case, it is shown that the out-of-plane relative displacement Δ*u_A_* is marginally affected, as for its period of vibrations. On the other hand, the vibrations following the period of strikes against the die surface are affected in amplitude, with obvious larger oscillations related to higher ***Q*** values; indeed, in this regard the effect of ***Q*** is not enough to close the gap between numerics and experimental. In the high-*g* case, values of ***Q*** higher than 10 lead to very small damping effects on sensor dynamics; hence, Δ*u_A_* keeps oscillating and the plate continuously strikes the die surface all over the investigated time period.

**Figure 11.**
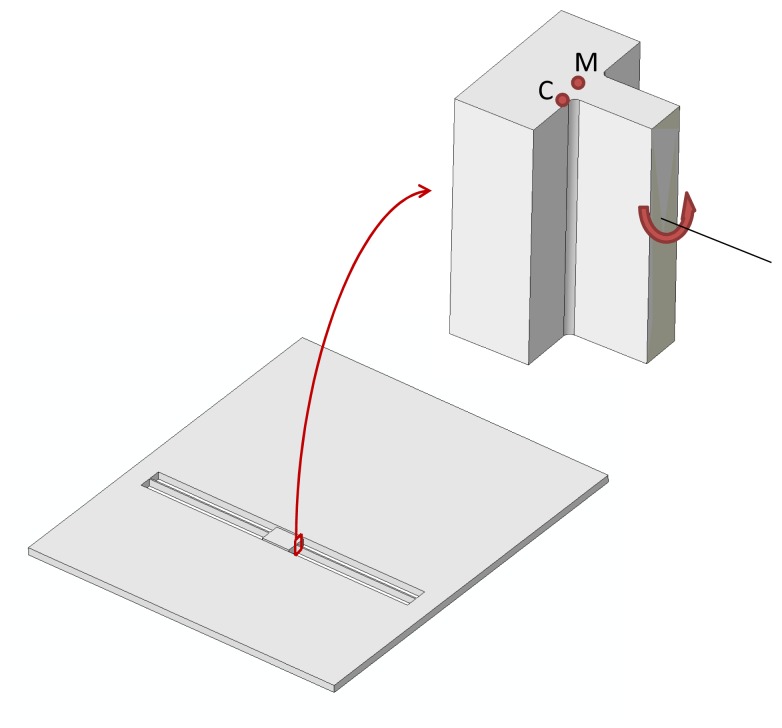
Model of the plate-spring connection region subjected to a torque, as adopted for the calculation of the stress intensity factor at the re-entrant corners.

Third, we assess the underestimation of peak values of *σ_P_* provided by the ROM. To account for the local stress intensification induced by the re-entrant corners and by the dynamics of the suspension springs not captured by the two fundamental deformation modes (torsional and out-of-plane bending ones) considered in the ROM, we provide this simple rationale. Since the intensification occurs only locally, like in fracture mechanics, and does not propagate much along the spring longitudinal axis, a small portion of the sensor is modelled, see Figure 11. By running a static FE analysis, with constrained anchor and under a torque applied to the suspension spring, the ratio between the maximum principal stresses at point C and at point M is computed as $\varphi_{P}=\frac{\sigma_{P}^{C}}{\sigma_{P}^{M}}\approx 5.5$. Here point C is chosen to be located at the root of one re-entrant corner, whereas point M is located along the longitudinal plane, where the theory of elasticity provides the computed stress levels given above, see [32]. This approach furnishes a simplified estimation of the stress intensification, since it does not account for inertial effects that prove different under different loading conditions. Such approximation leads to the results reported in Table 2, where peak values of *σ_P_* are compared, as obtained by FE analyses, by ROM simulations and by enhancing the ROM simulations through the amplification factor *φ_P_*. Table 2 shows that the so-called corrected ROM stress estimation is higher than the FE value in the low-*g* case, and only slightly smaller than the FE value in the high-*g* case.

## 4. Conclusions

To speed up the assessment of the reliability of a uni-axial inertial MEMS sensor under shock/drop loading conditions, we have provided and discussed a reduced order model of the movable parts of the sensor itself. We termed this model “physically-based”, since within a purely mechanical framework we accounted for the physics of the actually excited torsional and bending deformation modes of the sensor springs supporting the seismic, massive plate.

We have assessed the capability of the model and its accuracy against experimental data collected in a former laboratory campaign and also against three-dimensional finite element simulations. The second comparison looks necessary to check the accuracy of the model, due to the nonlinear dynamics induced by impacts featuring acceleration peaks much beyond the working range (in our case, ±2 *g*) and by the contact conditions with die and cap surfaces defining the sensor cavity. This requires a comparative assessment on the basis of local quantities, like, e.g., the out-of-plane displacement of the plate and the stress field in the suspension springs. The device output voltage is eventually adopted to compare the two numerical approaches with the experimental outcomes.

It has been shown that the reduced order model provides rather accurate estimations of sensor dynamics, up to acceleration peaks in the order of 5, 000 *g*. The only issue evidenced by the results is linked to the stress field in the suspension springs, which is underestimated by the proposed model. Such discrepancy is caused by the local stress intensification caused by the re-entrant corners at the end cross-sections of the springs, where they are connected to the anchor or the seismic plate. In these regions, prone to fail by cracking in case of extreme high-*g* shocks, the beam kinematics adopted to describe the system behaviour and to compute the stress field, does not hold true and corrective factors should be adopted. We have reported a proposal to define the corrective factor relevant to stress intensification, and showed that it allows to better match the finite element results. Next step of the present investigation will be a multiscale-like coupling between the proposed reduced order model of the whole sensor and detailed analyses in the critical regions, wherein brittle or quasi-brittle cracking can be incepted as soon as the tensile strength of polysilicon is attained. This approach is expected to further increase the accuracy of meso-scale analyses at the sensor level, which are necessary from a computational side of reliability analysis of inertial micro-sensors.

## Figures and Tables

**Table 1.** Computational gain, given by the ratio between the FE CPU time and the ROM CPU time.

	Speedup factor
Low-*g* test	725
High-*g* test	53

**Table 2.** Test-induced maximum values of the principal stress *σ_P_*. Comparison among: FE results, ROM outcomes, and ROM results corrected through the model-specific stress intensity factor (see Figure 11).

	FE (MPa)	ROM (MPa)	Corrected ROM (MPa)
Low-*g*	13.23	4.22	23.21
High-*g*	33.54	5.12	28.16
